# Pattern of contraceptive use among reproductive-aged women with diabetes and/or hypertension: findings from Bangladesh Demographic and Health Survey

**DOI:** 10.1186/s12905-022-01822-x

**Published:** 2022-06-15

**Authors:** Md Nuruzzaman Khan, M. Mofizul Islam, Rakibul M. Islam

**Affiliations:** 1 Department of Population Science, Jatiya Kabi Kazi Nazrul Islam University, Trishal Mymensingh, 2220 Bangladesh; 2 Department of Public Health, La Trobe University, Melbourne, Australia; 3 School of Public Health and Preventive Medicine, Monash University, Melbourne, Australia; 4 South Asian Institute for Social Transformation (SAIST), Dhaka, Bangladesh

**Keywords:** Diabetes, Hypertension, Contraception, Bangladesh

## Abstract

**Background:**

The prevalence of chronic conditions such as diabetes and hypertension is increasing among reproductive-aged women in Bangladesh. However, the pattern of contraceptive use among this population remains unknown. We, therefore, explored the pattern of contraceptive use among reproductive-aged women with diabetes and/or hypertension in Bangladesh.

**Methods:**

We extracted and analysed data of 3,947 women from the 2017/18 Bangladesh Demographic and Health Survey. Women’s pattern of contraceptive use was our outcome variable. We first classified the contraceptive using status as no method use, traditional method use (periodic abstinence, withdrawal, other traditional) and modern method use (pill, intra-uterine device, injections, male condom, female sterilization, male sterilization). We later classified these as (i) no contraceptive use vs any contraceptive use, (ii) traditional method or no use vs modern method use, (iii) traditional method vs modern method use. The explanatory variables were diagnosis of diabetes only, hypertension only or both diabetes and hypertension. The multilevel Poisson regression with robust variance was used to explore the associations.

**Results:**

The overall prevalence of contraceptive use was 68.0% (95% CI 66.3–69.7). The corresponding prevalences were 69.4% (95% CI 61.8–76.1) in women with diabetes only, 67.3% (95% CI 63.5–70.9) with hypertension only, and 62.0% (95% CI 52.8–70.4) in women having both diabetes and hypertension. The prevalence of modern methods of contraceptive use was lower (46.4%, 95% CI 37.4–55.6) and traditional methods use was higher (16.6%, 95% CI 13.8–16.8) in women who had both diabetes and hypertension than in women who did not have these conditions. The fully adjusted regression model showed that the prevalence of traditional method use was 31% (Prevalence ratio: 1.31, 95% CI 1.02–2.01) higher in women having both diabetes and hypertension compared with their counterparts who had none of these conditions.

**Conclusion:**

In Bangladesh, women with both diabetes and hypertension were more likely to use traditional contraception methods. These women are likely to experience increased risks of unwanted pregnancies and associated adverse maternal and child health outcomes. Targeted policies and programs should be undertaken to promote modern contraceptive use among women living with both chronic conditions.

## Introduction

Chronic conditions such as diabetes and hypertension are rising in low- and middle-income countries (LMICs), including Bangladesh [1–3]. This rising pattern is even more evident among reproductive-aged women (15–49 years) due to an increasing prevalence of overweight and obesity in younger women [2, 3]. As overweight and obesity are major predictors of being diabetic and hypertensive [4], there is a risk of further rise of diabetes and hypertension among reproductive-aged women in the coming years.

Women with diabetes and hypertension are at increased risk of pregnancy-related complications, including pre-eclampsia, preterm labour, polyhydramnios, and increased operative deliveries [5]. They are also more likely to give birth to babies with congenital anomalies and stillbirths [6]. Moreover, both prediabetes and hypertension are risk factors for gestational diabetes [7, 8]. Studies showed that offspring whose mothers have gestational diabetes are at increased risk of high blood pressure during early childhood [9, 10]. There is also evidence that offspring of mothers with gestational diabetes are predisposed to metabolic syndrome and its components [11–13], a pathway to long-term disease burden. Because of these adverse pregnancies and birth outcomes, delaying pregnancies up to the time when mothers controlled these conditions is of utmost importance [14]. Thus, there is an urgency to ensure effective family planning and contraceptive use along with proper management of diabetes and hypertension.

A limited number of studies explored the pattern of contraceptive use among women with diabetes and hypertension in LMICs [15–22], and the findings are inconclusive. The reasons for variation in these findings include the differences in study designs and sample sizes as well as the number of chronic conditions included as explanatory variables[15–22]. Other notable reasons are country-level variation in contraceptive use and difference in policies and programs to deliver contraceptives as part of health care services to manage diabetes and hypertension [22]. Also, pattern of contraceptive use among women with diabetes and hypertension were not compared with women without these chronic conditions [20]. These studies, therefore, offer limited information for formulating population-specific policies and programs for promoting contraception.

Previous studies in Bangladesh and other LMICs reported inadequate management of diabetes and hypertension and a relatively high prevalence of unintended pregnancies (47%) due to using no contraceptives or less effective methods [2, 3, 23–27]. However, there are little data in LMICs, including Bangladesh, about contraceptive use pattern among women with diabetes and/or hypertension. We, therefore, explored the pattern of contraceptive use in reproductive-aged women with and without diabetes and/or hypertension in Bangladesh.

## Materials and methods

### Study design

We analysed data from the most recent Bangladesh Demographic and Health Survey (BDHS) conducted in 2017/18. The survey was conducted by the DHS program of the USA. The National Institute of Population Research and Training of the Ministry of Health and Family Welfare of Bangladesh worked as a local partner. The survey followed a two-stage stratified random sampling method. In the first stage, 675 primary sampling units (PSUs) were selected randomly from a list of 293,579, which were created as part of the most recent Bangladesh Population and Housing Census. However, the survey could not be conducted in three PSUs due to extreme flooding during the data collection. In the second stage, 20,160 households were selected for data collection, with 30 households chosen from each selected PSU. Of them, the interview was completed in 19,457 households with a 96.5% inclusion rate. There were 20,376 eligible women in these selected households, and data were collected from 20,127 women. Informed consent was obtained from all participants. The contraception data was collected from each of these women. However, diabetes and hypertension-related data were collected from one-fourth of the selected households (7 to 8 households per PSU). Unlike contraception data, diabetes and hypertension data were collected from both males and females aged 18 years and more in the selected households. There were 14,704 respondents in these selected households, 12,924 of them (men: 5,583; women: 7,341) had blood pressure measured and blood glucose tested. Details of the sampling procedure have been published elsewhere [28].

### Analytic sample

Data of 3,947 women who met the following inclusion criteria were analysed in this study. The inclusion criteria were: (i) reproductive-aged fertile women who were not pregnant or not in the post-partum amenorrhea period at the time of data collection and (ii) reported their blood pressure and blood glucose or their medication status to control blood pressure and/or blood glucose.

### Outcome measures

Based on women’s contraceptive use status (yes, no) and the type of methods they used (e.g., pill, condom, sterilization), we generated three different outcome measures: (i) no contraceptive use vs any contraceptive use (modern or traditional method), (ii) traditional method or no use vs modern method use, (iii) traditional method vs modern method use.

### Explanatory variables and confounders

Women’s diabetes and hypertension status were our main explanatory (independent) variables classified as none, diabetes only, hypertension only, and both diabetes and hypertension. Confounders were considered at the individual, household, and community-level factors based on previous relevant literature [2–4, 15, 17, 29]. Individual-level factors were women's age (≤ 19 years, 20–34 years, ≥ 35 years), education (no education, primary education, secondary education, higher education) and body mass index (BMI: underweight, normal weight, overweight, obese). We followed the World Health Organization (WHO) recommendation for the Asian population’s body mass index to classify women's BMI in this study [30]. Husband’s education (no formal education, primary education, secondary education, higher education), number of children ever born (no child, 1–2 children, > 2 children) and wealth quintile (poorest, poorer, middle, richer, richest) were the household level factors. The BDHS created the wealth quintile variable using the principal component analysis of women’s responses on the households’ durable goods such as materials to build houses and the ownership of radio/television. Women’s place of residence (urban, rural) and administrative divisions (Barishal, Chattogram, Dhaka, Khulna, Mymensingh, Rajshahi, Rangpur, Sylhet) were community-level factors.

### Statistical analysis

We reported frequency and percentage to describe the characteristics of the respondents. The prevalence of contraceptive use in general and across diabetes and hypertension status of the women were calculated and reported with 95% confidence intervals (95% CI). We used multilevel mixed-effect Poisson regression with robust variance to estimate the associations between the outcomes and explanatory variables. We used Poisson regression because the odds ratio using logistic regression in cross-sectional studies may significantly overestimate the effect size when the outcomes are common (e.g., prevalence > 10%) [31, 32]. Secondly, in the dataset, individuals were nested within households; and households were nested within PSUs. This nested data structure generated multiple hierarchies and dependencies. The multilevel mixed-effects Poisson regression accounts for these multiple hierarchies and dependencies in data and the problem of overestimation [33]. We developed unadjusted and adjusted models (adjusted for individual, household, and community level confounders) for each of the three study outcomes. For instance, the first set of models examined the associations between women’s status of chronic conditions and contraceptive use (no use vs any methods use). The second set examined the associations between women’s status of chronic conditions and contraceptive use type (traditional methods/no use vs modern methods use). The third set examined the association with contraception method type (traditional methods vs modern methods use). Sampling weight and complex survey design were considered in all analyses. Results are reported as prevalence ratio (PR) with its corresponding 95% CI. Multicollinearity and interactions were checked. All statistical tests were two-sided, and a *p*-value < 0.05 was considered statistically significant. The study was designed and reported following the Strengthening the Reporting of Observational Studies in Epidemiology (STROBE) guidelines [34]. All methods were performed in accordance with the relevant guidelines and regulations. All analyses were performed using the statistical software package Stata (version 15.1; Stata Corp LP, College Station, TX, USA).

## Results

The study included data from 3,947 women of reproductive age. Participants’ mean (standard deviation) age was 33 (8.86) years (Table 1). Around 20% of women did not receive formal education and 3% were nulliparous. At the time of the survey, over three-fourth of women were living in rural areas. Around half of the women were overweight/obese, 19.9% were hypertensive, 5.2% had diabetes and 3.6% had both diabetes and hypertension.

**Table 1:** Characteristic of women of reproductive age 15–49 (n = 3947)

Characteristics	Frequency (%)
Age in years, mean (standard deviation)	33.0 (8.86)
15–19	231 (5.86)
20–34	1992 (50.46)
35–49	1724 (43.67)
Level of education	
No education	779 (19.75)
Primary	1326 (33.60)
Secondary	1440 (36.50)
Higher	401 (10.15)
Body mass index (kg/m^2^)	
Underweight	1517 (38.50)
Normal weight	463 (11.76)
Overweight	1362 (34.48)
Obese	605 (15.27)
Husband’s education	
No formal education	903 (24.66)
Primary	1218 (33.26)
Secondary	1051 (28.71)
Higher	489 (13.36)
Wealth quintile	
Poorest	789 (19.99)
Poorer	831 (21.07)
Middle	825 (20.89)
Richer	757 (19.17)
Richest	745 (18.89)
Number of children ever born	
No child	119 (3.02)
1–2 children	1942 (49.19)
> 2 children	1886 (47.80)
Place of residence	
Urban	1036 (26.25)
Rural	2911 (73.75)
Administrative divisions	
Barishal	225 (5.69)
Chattogram	675 (17.11)
Dhaka	931 (23.60)
Khulna	494 (12.52)
Mymensingh	300 (7.59)
Rajshahi	592 (15.00)
Rangpur	501 (12.69)
Sylhet	229 (5.80)
Chronic conditions	
None	2811 (71.20)
Diabetes only	206 (5.24)
Hypertension only	786 (19.92)
Both diabetes and hypertension	144 (3.64)

The prevalence of contraceptive use by women with diabetes and/or hypertension is presented in Table 2. Around 68.0% (95% CI 66.3%—69.7%) of women reported using modern or traditional contraceptives. This percentage was 62.0% (95% CI 52.8%—70.4%) among women who had both diabetes and hypertension, and went down further when examined separately for modern contraceptive methods use (46.4%, 95% CI 37.4%—55.6%). The prevalence of modern contraceptive use was 56.4% (95% CI 48.5%—63.9%) among women with diabetes, 54.4% (95% CI 50.3%—58.4%) among women with hypertension and 46.4% (95% CI 37.4%—55.6%) among women who had both diabetes and hypertension.

**Table 2:** Pattern of contraceptive use in Bangladesh, BDHS 2017–2018

Contraceptive methods	Overall	Prevalence of contraceptive use (95% CI)			
		None of diabetes and hypertension	Diabetes only	Hypertension only	Both diabetes and hypertension
**Any method**	**68.0 (66.3–69.7)**	**68.4 (66.4–70.4)**	**69.4 (61.8–76.1)**	**67.3 (63.5–70.9)**	**62.0 (52.8–70.4)**
**Modern methods**	**56.96 (55.1–58.8)**	**58.3 (56.2–60.4)**	**56.4 (48.5–63.9)**	**54.4 (50.3–58.4)**	**46.4 (37.4–55.6)**
Oral contraceptive pills	27.2 (25.6–28.9)	27.6 (25.7–29.5)	18.5 (13.3–25.3)	29.1 (25.7–32.7)	21.3 (14.3–30.6)
Injectables	12.4 (11.2–13.7)	13.1 (11.7–14.7)	13.1 (8.7–19.3)	10.7 (8.6–13.4)	7.3 (3.8–13.8)
Condoms	7.5 (6.5–8.6)	8.0 (6.9–9.3)	11.8 (7.7–17.6)	4.4 (3.1–6.1)	7.8 (4.4–13.7)
Female sterilization	5.6 (4.7–6.5)	4.8 (4.0–5.8)	5.5 (2.9–10.1)	7.8 (5.8–10.3)	8.5 (4.7–14.9)
Male sterilization	1.2 (0.9–1.7)	1.2 (0.8–1.8)	2.5 (1.1–5.8)	1.2 (0.6–2.2)	0.3 (0.004–1.9)
Intra Uterine Device	0.6 (0.4–1.0)	0.7 (0.4–1.1)	1.5 (0.4–4.7)	0.4 (0.01–1.5)	0
Implants	2.5 (2.0–3.1)	2.9 (2.3–3.7)	3.5 (1.4–8.0)	0.9 (0.4–2.0)	1.1 (0.2–7.4)
**Traditional methods**	**11.1 (9.1–12.4)**	**10.2 (9.2–11.6)**	**13.0 (11.9–14.5)**	**12.9 (10.8–13.9)**	**15.6 (13.8–16.8)**
Periodic abstinence	7.8 (7.0–8.8)	7.0 (6.1–8.1)	10.7 (7.0–16.0)	9.3 (7.4–11.7)	11.3 (7.0–17.7)
Withdrawal	2.9 (2.3–3.5)	2.8 (2.3–3.6)	2.2 (0.8–5.6)	2.9 (1.9–4.5)	4.3 (1.8–10.1)
Other traditional methods	0.4 (0.2–0.7)	0.3 (0.1–0.7)	0.2 (0.002–1.5)	0.7 (0.3–1.6)	0
**Currently not using**	**32.0 (30.3–33.7)**	**31.6 (29.6–33.6)**	**30.6 (23.9–38.2)**	**32.7 (29.1–36.5)**	**38.0 (29.63–47.2)**

World Health Organization’s recommendation was used to group contraceptive methods.

Among modern contraceptives, oral contraceptive pills (27.2%, 95% CI 25.6%—28.9%) and injectables (12.4%, 95% CI 11.2%—13.7%) were dominant methods in all women as well as in women with diabetes only, hypertension only, or both diabetes and hypertension. The use of traditional contraceptive methods increased from 11.1% (95% CI 9.1%—12.4%) among all women to 15.6% (95% CI 13.8%—16.8%) among women who had both diabetes and hypertension.

Unadjusted and adjusted associations between contraceptive use and diabetes and/or hypertension generated from multiple mixed-effect Poisson regression models are reported in Table 3. Unadjusted models show that women with both diabetes and hypertension were significantly more likely than women who had none of these conditions to report using traditional methods or no contraceptives (PR: 1.29, 95% CI 1.08–1.53; *p* = 0.005). Women who had hypertension only (PR: 1.30, 95% CI 1.04–1.62; *p* = 0.021) or had both diabetes and hypertension (PR: 1.70, 95% CI 1.14–2.52; *p* = 0.008) were significantly more likely than their counterparts to report using traditional methods of contraception.

**Table 3:** Unadjusted and adjusted associations between types of contraceptives use and women with diabetes and/or hypertension

Chronic conditions	Contraceptive methods					
	No use *vs* any methods use		Traditional methods or no use *vs* modern methods use		Traditional methods *vs* modern methods use	
	Unadjusted PR (95% CI) *p *value	Adjusted^*^ PR (95% CI) * p *value	Unadjusted PR (95% CI) * p *value	Adjusted^*^ PR (95% CI) * p *value	Unadjusted PR (95% CI) * p *value	Adjusted^*^ PR (95% CI) * p *value
None	1.00	1.00	1.00	1.00	1.00	1.00
Diabetes only	0.98 (0.76–1.23)* p* = 0.785	0.88 (0.67–1.15)* p* = 0.340	1.05 0(0.87–1.25)* p* = 0.625	0.95 (0.78–1.15)* p* = 0.568	1.27 (0.86–1.85)* p* = 0.225	1.08 (0.69–1.54)* p* = 0.685
Hypertension only	1.04 (0.91–1.18)* p* = 0.603	0.99 (0.84–1.15)* p* = 0.744	1.09 (0.99–1.21)* p* = 0.079	%1.%2(0.90–1.13)* p* = 0.991	**1.30 (1.04–1.62)*** p* = **0.021**	1.04 (0.84–1.30)* p* = 0.706
Both diabetes and hypertension	1.20 (0.95–1.52)* p* = 0.122	%1.%2(0.75–1.36)* p* = 0.960	**1.29 (1.08–1.53)*** p* = **0.005**	1.08 (0.88–1.34)* p* = 0.462	**1.70 (1.14–2.52)*** p* = **0.008**	**1.31 (1.02–2.01)*** p* = **0.005**

PR = Prevalence ratio; CI = Confidence Interval

*Models were adjusted for women’s age, education, body mass index, husbands’ education, wealth quintiles, number of children ever born, place of residence and administrative divisions

When models were adjusted for women’s age, education, BMI, husbands’ education, wealth quintiles, number of children, place of residence and administrative divisions, most of the associations reported in the unadjusted disappeared except for the traditional contraceptive use. We found a 31% (aPR: 1.31, 95% CI: 1.02–2.01) higher prevalence of traditional contraceptive methods use among women who had both diabetes and hypertension compared with women who did not have diabetes or hypertension.

## Discussion

In this study of a nationally representative sample of 3,947 reproductive-aged women, the prevalence of modern and traditional contraceptive use was 58% and 30%, respectively. The use of modern contraceptives was lower while traditional and non-use of contraception were higher in women with diabetes and/or hypertension. Following the adjustment of individual-, household-, and community-level factors, the prevalence of traditional contraceptive method use was 31% higher in women who had both diabetes and hypertension compared with women who had none of these conditions. To the best of our knowledge, this is a novel finding for Bangladesh and other LMICs. As traditional contraceptive methods increase the risk of unintended pregnancy, adverse pregnancy and birth outcomes, these study findings have policy implications about ensuring the use of modern contraception methods in women with chronic conditions, especially those who have both diabetes and hypertension.

Although our study found a relatively low prevalence of hypertension (20%) and diabetes (5%) compared to the national prevalence (27.4% and 9.8%, respectively) among women [2, 3], the rate of diabetes and hypertension in women of reproductive age is gradually increasing in Bangladesh [2, 3, 28]. These chronic conditions have a range of adverse consequences; for instance, hypertensive disorders are the third major cause of maternal mortality in LMICs [35]. The consequences could be aggravated further if women have both diabetes and hypertension and much of these adverse consequences are preventable by ensuring planned pregnancies [14]. However, this is quite challenging in Bangladesh and other LMICs, because of the low prevalence of contraceptive use, financial difficulties, and misunderstanding and/or misconception associated with pregnancies among women with diabetes and/or hypertension [20, 24]. Well-designed programs are needed for effective management of diabetes and hypertension along with appropriate family planning services to delay pregnancies until women are ready for conception [36].

Around 32% of women in our study did not use contraception and 11% depended on traditional contraceptive methods. Previous studies identified several reasons for such high levels of traditional contraceptive use or no contraceptive use, including (i) lack of awareness of the importance of contraceptive use, (ii) difficulties in accessing contraceptives from government-run sources, and (ii) financial difficulties in accessing contraceptives from private sources, or combinations of these factors [24, 37]. Religious directives that Islam (religion of over 90% of people in Bangladesh) does not permit contraceptive use is also a common barrier to the access and use of contraception [24]. The cumulative effects could worsen the situation for women with diabetes and hypertension, as the findings of our study suggest. The effective management of diabetes and hypertension needs continuous treatment, medication and visits to healthcare facilities [38]. These requirements can create economic burdens because management care for diabetes and hypertension is mainly available at private healthcare facilities, which are generally expensive [3]. Additional expenses associated with modern contraceptive use could be a further barrier. Moreover, the healthcare facilities that provide care for the management of diabetes and hypertension are usually separate from the healthcare facilities that deliver family planning services [39]. Women may not receive counselling for contraceptive use due to the exclusive focus on the management of diabetes and hypertension [39]. These fragmented services affect women’s knowledge of the importance of using effective contraception and the risk of becoming pregnant until they have controlled chronic conditions. Bangladesh should prioritize initiatives to ensure contraception counselling and distribution as part of diabetes and hypertension management care [40–42].

Literature suggests that selective modern contraceptive methods, e.g. progestin-only contraceptives and condom use, are suitable for women with diabetes and hypertension [22]. However, misunderstandings are common; for instance, a significant percentage of women generally believe that contraceptive use elevates blood glucose and blood pressure [43–46]. Another misapprehension is that chronic diseases may reduce women’s fertility, and thereby effective contraceptive use is considered unnecessary [15, 47]. Intrauterine and vaginal devices are considered risky for women with diabetes and hypertension, as they need to be inserted through surgical operation. Moreover, women with chronic conditions who desire pregnancy may not be sure of the appropriate timing because of their inadequate knowledge of whether conception would put them at risk [15, 17]. This ambivalence about timing is associated with no use or the use of traditional contraceptive methods [24]. It is also possible that women with uncontrolled diabetes and hypertension are sexually less active and thus do not use contraceptives [20]. Besides, as there are some potential risks of using hormonal contraception in certain patients with metabolic syndrome, fear of exacerbating the disease may prevent some clinicians from recommending contraception [48]. Moreover, one potential misunderstanding among many providers is that women with a medical condition cannot take estrogen [49], which might be conducive in Bangladesh. These misunderstandings are additional for the women with diabetes and hypertension to the common community level general misunderstandings about contraception use, e.g. contraception use can cause uterine fibroids, cancer, infertility, and birth defects [50]. Our results could be a confluence of all these factors. While further research is needed to identify the reasons for low use of modern contraceptive, our findings suggest the need for targeted policies and programs to promote appropriate contraceptive use by women with chronic conditions.

The current study has several strengths. We analyzed a large nationally representative dataset and thus our findings are likely to be generalisable in Bangladesh and other countries of similar features. Also, diabetes and hypertension were measured using high-quality techniques and we classified them following the WHO guidelines. The BMI was classified following the WHO recommendation for the Asian population. The analytical approach we used considered the hierarchical structure of the data and avoided the chance of effect-size overestimation that may occur if conventional logistic regression is employed in cross-sectional studies. We adjusted the regression models for a wide range of potential confounding factors. This study also has some limitations. As we analysed cross-sectional data, the findings are correlational only. Another limitation was possible reporting bias in diabetes and hypertension status, which were self-reported. However, any such reporting error is likely to be random. We recommend prospective cohort data for future studies of contraceptive use in women with chronic conditions, including diabetes and hypertension.

## Conclusion

In Bangladesh, women with both diabetes and hypertension were more likely to use traditional contraceptive methods. These women are likely to experience increased risks of unwanted pregnancies and associated adverse maternal and child health outcomes. Targeted policies and programs should be undertaken to promote modern contraceptive use among women diagnosed with both of these chronic conditions. Contraception should also be included in diabetes and hypertension care management for reproductive-aged women.

## Data Availability

The data that support the findings of this study are available from The DHS Program, but restrictions apply to the availability of these data, which were used under license for the current study, and so are not publicly available. Data are, however, available from the authors upon reasonable request and with permission of The DHS Program (https://dhsprogram.com/data/).

## Abbreviations

LMICs: Low- and middle-income countries
BDHS: Bangladesh Demographic and Health Survey
NIPORT: National institute of population research and training
WHO: World Health Organization
PR: Prevalence ratio
PSU: Primary sampling unit
